# Effect of Assembly Stresses on Fatigue Life of Symmetrical 65Si7 Leaf Springs

**DOI:** 10.1155/2014/762561

**Published:** 2014-08-04

**Authors:** Vinkel Kumar Arora, Gian Bhushan, M. L. Aggarwal

**Affiliations:** ^1^Department of Mechanical Engineering, ITM University, Sector 23A, Gurgaon, Haryana 122017, India; ^2^Department of Mechanical Engineering, National Institute of Technology, Kurukshetra, Haryana 136019, India; ^3^Department of Mechanical Engineering, YMCA University of Science &Technology, Faridabad, Haryana 121006, India

## Abstract

The maximum stress induced plays vital role in fatigue life improvement of leaf springs. To reduce this maximum stress, leaves with different unassembled cambers are assembled by pulling against each other and a common curvature is established. This causes stress concentration or sets assembly stress in the assembled leaf springs which is subtractive from load stress in master leaf while it is additive to load stress for short leaves. By suitable combination of assembly stresses and stepping, it is possible to distribute the stress and improve the fatigue life of the leaf spring. The effect of assembly stresses on fatigue life of the leaf spring of a light commercial vehicle (LCV) has been studied. A proper combination of stepping and camber has been proposed by taking the design parameters into consideration, so that the stress in the leaves does not exceed maximum design stress. The theoretical fatigue life of the leaf springs with and without considering the assembly stresses is determined and compared with experimental life. The numbers of specimens are manufactured with proposed parameters and tested for load rate, fatigue life on a full scale leaf springs testing machine. The effect of stress range, maximum stress, and initial stress is also discussed.

## 1. Introduction

Leaf springs are most frequently used in the suspensions. Leaf springs like all other springs serve to absorb, store, and release the energy. In heavy vehicles, leaves are stacked one upon another to ensure rigidity and strength. During its operation, the leaf spring requires that the load rate should vary within limit (±7%) and the maximum stress induced should be lower than the maximum design stress. For a given stress range the leaf spring should have maximum fatigue life or as specified. The stress range and the maximum stress induced in the leaf spring play a vital role in deciding the load rate and fatigue life of the leaf spring. The maximum stress induced can be reduced by assembling the leaves with different radii of curvature and establishing a common curvature under no load. The proper distribution of the stress between the leaves can enhance the fatigue life of the leaf springs.

Aggarwal et al. [1] evaluated the axial fatigue strength of EN45A spring steel specimen experimentally as a function of shot peening in the conditions used. *S*/*N* curves of the specimens were correlated with leaf springs curve in vehicles. Aggarwal et al. [2] concluded that the influence of high contact pressure and temperatures resulted in microweld between the two leaf surfaces. The fatigue strength of the leaf springs was studied as a function of shot peening parameters. Saelem et al. [3] simulated a leaf springs model. An experimental leaf springs model was verified by using a leaf springs test rig that could measure vertical static deflection of leaf springs under static loading condition. The results showed a nonlinear relationship between the applied load and the leaf springs deflection for both directions of loading, in form of a hysteresis loop. Fuentes et al. [4] studied the origin of premature failure analysis procedures, including examining the leaf spring history. The visual inspection of fractured specimens and simulation tests on real components were also performed. It was concluded that fracture occurred by a mechanism of mechanical fatigue initiated at the region of the central hole, which suffered the highest tensile stress levels. Kumar and Vijayarangan [5] described static and fatigue analysis of steel leaf springs and composite multileaf springs made up of glass fibre reinforced polymer using life data analysis. The dimensions of an existing conventional steel leaf spring of a light commercial vehicle were taken and verified by design calculations. Static analysis of 2D model of conventional leaf springs was also performed using ANSYS 7.1, and the results obtained were compared with experimental results. Patunkar and Dolas [6] worked on nonlinear force displacement of each leaf spring as well as the spring characteristics of a pack consisting of two to four leaves using ANSYS. The results from ANSYS were compared with those from the test, which showed a fairly good agreement with each other.

The objective of the present work is to determine the effect of assembly stresses on fatigue life reliability of leaf springs. It is available in open literature that with proper combination of stepping and individual leaf camber the stress distribution can be uniform along the leaf. The 65Si7 leaf springs design parameter of a light commercial vehicle is taken into consideration for this work. This paper is divided into two parts. In part one, a design procedure for determination of total moment of inertia, number of leaves, and stepping and individual leaf camber is established. The stepping and individual leaf camber is proposed with a view to lower stresses. Using SAE approach fatigue life of the leaf spring is determined theoretically by considering and not considering the assembly stresses. In part two, three leaf springs specimens lots (four leaf spring assemblies in each lot) have been manufactured. All the specimens are tested on a full scale leaf spring testing machine for fatigue life of the leaf spring. The theoretical and experimental fatigue life results (with and without assembly stresses) are compared for validation. The effect of assembly stresses and stress range on fatigue life is also depicted.

## 2. Material

The 65Si7/SUP9 grade material is used for the experimental work. The chemical composition of the material is shown in Table 1.

The mechanical properties and parameters of the 65Si7 are shown in Table 2. The material is heat-treated at 880°C and oil-quench-hardened and it is tempered at 410°C for 90 minutes to get tempered martensite structure.

## 3. Leaf Spring Design Parameters

A leaf spring is considered as a beam of uniform strength composed of leaves of equal thickness where the fiber stress is the same throughout the length of the beam. This approximation is justified for most of the springs within the accuracy necessary for layout work and with certain correction factors for estimate of required length, overhang, camber, width, thickness, and number of leaves. The parameters are categorized as design parameters which are indicated in Table 3.

## 4. Analytical Method for Static Analysis of Leaf Springs

**Figure 1:**
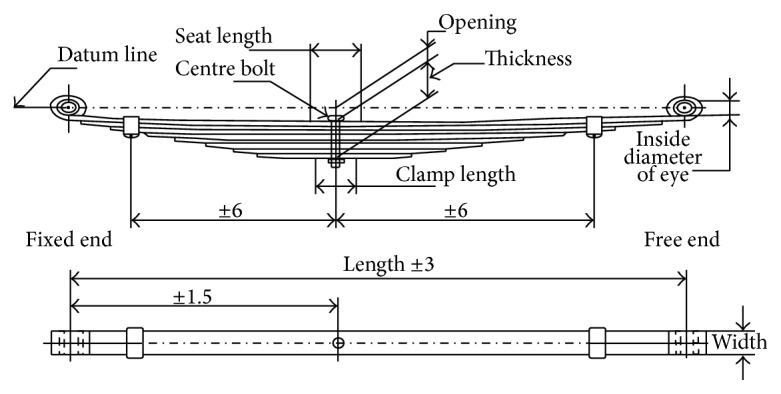
Leaf spring assembly layout drawing [7].

The leaf spring assembly layout is shown in Figure 1. The calculation of the leaf spring parameters involves certain number of steps. As per SAE spring design manual approach, the steps involved in leaf springs design are as follows.

### 4.1. Total Moment of Inertia (Required for Desired Load Rate)

Consider

(1) $$\begin{array}{c} \sum I=\frac{k*L^{3}}{32*E*\text{SF}}=34351.7\;{\text{mm}}^{4}, \end{array}$$

where *k* = load rate, *L* = spring span, *E* = Young's modulus, and SF = stiffening factor.

### 4.2. Maximum Permissible Thickness for the Leaf Section

Consider

(2) $$\begin{array}{c} t_{\max}=\frac{8*\sum I*S_{\max}}{L*P_{\max}}=8.20\;\text{mm}. \end{array}$$

### 4.3. Determination of Number of Leaves

Consider

(3) $$\begin{array}{c} \sum I_{1}=N_{1}*i_{1}, \end{array}$$

where *N*_1_ = number of leaves with thickness *t*_1_,  *i*_1_ = moment of inertia for section *t*_1_ = 8 mm, and width *b* = 70 mm and

(4) $$\begin{array}{c} i_{1}=\frac{b_{1}^{\prime }*t_{1}^{3}}{12}+\frac{3.1428*t_{1}^{4}}{64}=2846.5\;{\text{mm}}^{4}, \end{array}$$

where *b*_1_′ = *b* − *t*_1_ = 62 mm and *N*_1_ = 11. Consider

(5) $$\begin{array}{c} \sum I_{1}=11*2846.13=31311\;{\text{mm}}^{4}\\ \sum I_{2}=N_{2}*i_{2}, \end{array}$$

where *N*_2_ = number of leaves with thickness *t*_2_, *i*_2_ = moment of inertia for section *t*_2_ = 7 mm, and width *b* = 70 mm and

(6) $$\begin{array}{c} i_{2}=\frac{b_{2}^{\prime }*t_{2}^{3}}{12}+\frac{3.1428*t_{2}^{4}}{64}=1918.6\;{\text{mm}}^{4}, \end{array}$$

where *N*_2_ = 1 and *b*_2_′ = *b* − *t*_2_ = 63 mm and

(7) $$\begin{array}{c} \sum I_{2}=1*1918.6=1918.6\;{\text{mm}}^{4}\\ \sum I_{\text{total}}=\sum I_{1}+\sum I_{2}=33230\;{\text{mm}}^{4}. \end{array}$$

### 4.4. Modified Load Rate

Consider

(8) $$\begin{array}{c} K_{\text{modified}}=\frac{\sum I_{\text{total}}}{\sum I}*k=153.91\;\text{N}/\text{mm}\\ \%\;\;\text{age}\;\text{variation}\;\text{of}\;k=3.271\%\;\left(\text{Hence acceptable}\right)\text{.} \end{array}$$

### 4.5. Maximum Stress Induced

Consider

(9) $$\begin{array}{c} S_{\max}=\frac{P_{\max}*L*t}{8*\sum I_{\text{total}}}=963.34\;\text{MPa}. \end{array}$$

### 4.6. Stress Distribution between the Leaves

The seat clamp is taken into consideration for stress calculation between the leaves. The inactive length in the seat for a spring without liners is estimated as distance between the outside edges of the clamp bolts which is 100 mm for this spring. The active length for both front and rear cantilever will be 50 mm less than the distance from centre of the eye to centre of an axle seat. The stress at 50 mm distance from the centre of axle seat can be calculated as the following:

(10) $$\begin{array}{c} \text{Front}\;\text{cantilever}\;\text{stress},\;S_{\text{fa}}=\frac{P_{a}*l_{a}*t}{2*\sum I_{t}}\\ \text{Rear}\;\text{cantilever}\;\text{stress},\;S_{\text{rb}}=\frac{P_{b}*l_{b}*t}{2*\sum I_{t}}. \end{array}$$

 Front cantilever (a) length = *l* = *L*/2 = 575 mm and rear cantilever (b) length = *l* = *L*/2 = 575 mm.
 Cantilever ratio *Y* = *a*/*b* = 1 (symmetric spring) and seat length SL = 100 mm.
 Active length of front cantilever *l*_*a*_ = (*l* − SL/2) = 525 mm.
 Active length of rear cantilever *l*_*b*_ = (*l* − SL/2) = 525 mm.
 Design load on front cantilever *P*_*a*_ = *P*/2 = 6479.5 N.
 Design load on rear cantilever *P*_*b*_ = *P*/2 = 6479.5 N.
 Maximum load on front cantilever *P*_*a*max⁡_ = *P*_max⁡_/2 = 14005 N.
 Maximum load on rear cantilever *P*_*b*max⁡_ = *P*_max⁡_/2 = 14005 N.
 As the leaf spring is symmetric cantilever ratio = *Y* = *a*/*b* = 1; therefore,
 
*l*_*a*_ = *l*_*b*_, *P*_*a*_ = *P*_*b*_ (at design load) and *P*_*a*max⁡_ = *P*_*b*max⁡_ (at maximum load).
 Also *S*_fa_ = *S*_rb_ = *S*_*d*_ (at design load) and *S*_fa_ = *S*_rb_ = *S*_*m*_ (at maximum load).

The total stress induced in the leaf spring is the summation of load stress and assembly stress:

(11) $$\begin{array}{c} S=S_{l}+S_{a}. \end{array}$$

The load stress is determined from the design and maximum load. If all the leaves are fitted with the common unassembled curvature, then the assembly stress is zero; but the leaves are fitted in such a manner that the radius of curvature goes on increasing from the master leaf to the last leaf. With the use of different individual leaf camber, the assembly stress can be added to or deducted from the load stress of the assembled spring to obtain desirable stress pattern in the leaf spring. The assembly stress is arbitrarily chosen except they must be selected in increasing order from the main leaf to shorter leaf such that ∑*S*_*a*_ *t* ^2^ = 0. A negative assembly stress in the main leaf is required to reduce the maximum stress induced in the master leaf to safe limits as the longitudinal and lateral forces are more in the master leaf. A negative assembly stress is provided in the main leaf so as to reduce the maximum stress to about 866 MPa. The longitudinal and lateral forces imposed on the main leaf and also its greater stress range are the reasons for reducing its bending stress. In view of the condition ∑*S*_*a*_ · *t* ^2^ = 0, several selections of deducted and added stresses for the individual leaves are analysed before deciding on the best arrangement as shown in Table 4.

### 4.7. Design for Individual Leaf Camber

In almost all leaf springs the unassembled curvatures *q*_*n*_ are different in the unassembled leaves. In assembly, a common (unloaded) curvature *q*_*o*_ is established which is variable along the spring even if the leaves are made of circular arcs [8]. The individual leaf curvature is calculated from common curvature as the folowing:

(12) $$\begin{array}{c} q_{n}=q_{o}-\frac{S_{a}}{E*y}. \end{array}$$

Curvature is called positive in the direction of increasing load and camber is conventionally positive in the opposite direction. Camber can be converted into curvature as

(13) $$\begin{array}{c} q_{o}=\text{Curvature}=\frac{-8*\text{camber}}{{\text{length}}^{2}}=0.000574669\;{\text{mm}}^{-1}. \end{array}$$

The relationship between free curvature, assembly curvature, and loaded curvature is given by

(14) $$\begin{array}{c} q_{\text{free}}+q=q_{\text{loaded}}. \end{array}$$

For flat leaf, the curvature is zero and it increases in the direction of load application; and for most of the springs the free curvature is negative. The no load camber requirement for this leaf spring assembly is 95 ± 4 mm. The analytical individual leaf camber is shown in Table 5.

### 4.8. Determination of Deflection and Bending Stress

In the analytical approach the deflection can be determined as force per unit load rate. For different values of the load the corresponding values of deflection can be achieved. The stresses induced at different loads can be determined by substituting the values of the load in the stress induced formula. The analytical results for load, deflection, and bending stress are shown in Table 6.

### 4.9. Fatigue Life Estimation as per SAE Spring Design Manual Approach

Fatigue life is expressed by the number of deflection cycles a spring will withstand without failure or permanent set. A leaf spring used in a suspension will undergo a large number of cycles of small amplitude near the design load position without failure. Under the greater amplitude the number of cycles without failure will be reduced since the maximum stresses as well as the stress range are increased and both are determining factors in fatigue life of the spring. As per SAE spring design manual, this criteria is frequently used for determination of approximate fatigue life of the spring; initial stress (horizontal scale) and maximum stress (vertical scale) are intersected to estimate the number of cycles the spring will withstand for given loading condition.

The fatigue test stroke for leaf spring without considering assembly stress is determined as the following [8]:

 deflection to design load = 12959/153.1 = 84.6 mm,
 maximum load = 28 KN,
 metal-to-metal clearance (compression stroke) = 94.6 mm,
 total deflection to maximum load = 182.9 mm,
 stress at metal-to-metal position = 969 MPa,
 stress rate = 969/182.9 = 5.29 MPa/mm,
 release stroke = 0.5 × 94.6 = 47.3 mm,
 fatigue test stoke = 47.3 + 94.6 = 141.9 mm,
 initial stress = 969 − (141.9 × 5.29) = 218.3 MPa.

The fatigue test stroke for leaf spring by considering assembly stress is determined as the following:

 deflection to design load = 12959/153.1 = 84.6 mm,
 maximum load = 28 KN,
 metal-to-metal clearance (compression stroke) = 94.6 mm,
 total deflection to maximum load = 182.9 mm,
 stress at metal-to-metal position = 885 MPa,
 stress rate = 885/182.9 = 4.83 MPa/mm,
 release stroke = 0.5 × 94.6 = 47.3 mm,
 fatigue test stoke = 47.3 + 94.6 = 141.9 mm,
 initial stress = 885 − (141.9 × 4.83) = 199.6 MPa.

## 5. Manufacturing of Leaf Springs

For the production of high strength leaf springs, the process is comprised of shearing, punching, heat treatment, hot cambering, shot peening, scragging, and testing for load rate and durability. The processing of the raw material plays a vital role in achieving the required load rate and fatigue life. After punching and shearing, the raw material is moved to the hardening furnaces for heat treatment. The structure of the raw material is partial austenite, and after quenching the structure is martensite, but after the tempering process the structure should be tempered martensite. The material is heated in the furnace in the temperature range of 880–910°C depending upon the cross section thickness and width. The spring steel is having the thickness of 8 mm and width of 70 mm which is heated at 880°C to achieve full austenite structure. Hot cambering of the spring is done in this state by passing through finger cambering tools followed by quenching in oil at temperature of 80°C. Tempering is done at a temperature of 410°C for 90 min slow cooling till the tempered martensite structure is achieved. Surface treatment like shot peening and graphite coating is done on each individual leaf. The final assembly is done by pulling all the leaf with a centre nut and bolt. The scragging of the assembly is done and the u clips are attached. The assembly is tested for load rate and fatigue life.

## 6. Experimental Setup

The 65Si7 leaf springs assembly consists of two full length leaves and ten graduated leaves, four rebound clips of mild steel, four shim pipes with four nut and bolts, four rivets, centre nut and bolt, and bush of bronze. The full scale testing of leaf springs was carried out in an electrohydraulic static component testing system. The laminated leaf springs were placed in a fixture simulating the conditions of a vehicle. The setup consists of a hydraulic power pack to give a hydraulic pressure of 20.6 MPa with a flow rate of 210 liters per minute (lpm), which was sent to a hydraulic actuator to operate at a frequency of 0.3 Hz with the displacement specified by the alternating load. This involves applying the axial load on the leaf springs and measuring the deflection and bending stress. The conventional leaf spring was tested under static load condition by using hydraulic static load ram for load application. Mounting of the leaf spring was done by keeping it in inverted manner on the test bed. Two eye ends were held in the clamping devices and load was applied from the top, at the center of leaf springs. To measure the load dial indicator was used, which was located beside the full scale testing machine and deflection was measured by strain gauges located at the clamping of the test rig. The springs were loaded from unladen load (i.e., 7.6 KN) to maximum load (i.e., 28 KN). The vertical deflection of the springs at the unladen load, design load, flat load, rubber touching load, and metal-to-metal contact or maximum load was recorded, respectively, as per the standard operating procedure prescribed [7].

The leaf springs were tested on a full scale testing machine under the unladen load, rated load, flat load, rubber touching load, and metal-to-metal load, and the corresponding deflection and stress values observed are shown in Table 7. The experiments were conducted twice and the mean value of the results was considered. Table 7 depicts the observed values of deflection and stress corresponding to the loads applied on the shorter leaf by a static hydraulic ram.

### 6.1. Standard for Fatigue Life Determination of Leaf Springs

**Figure 2:**
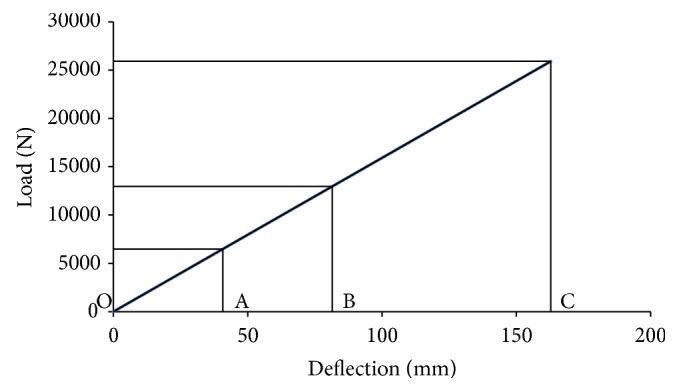
Load versus deflection for fatigue life determination.

As per the IS1135, the fatigue test is conducted in deflection. The spring shall be loaded from OA to OC as defined in Figure 2. Typically this can be between 0.5 times the rated load and twice the rated load unless otherwise specified by vehicle manufacturer [7]. Consider

(15) $$\begin{array}{c} \text{OA}=\text{OB}-\frac{\left(\text{OC}-\text{OB}\right)}{2}, \end{array}$$

where OA = load/deflection corresponding to rated load.

Consider OB = load/deflection corresponding to maximum load experienced under actual vehicle conditions typically 2 g, where g is the load shared by springs under the laden condition of the vehicle.

Consider OC = load/deflection corresponding to initial stress.

### 6.2. Experimental Fatigue Life Determination

**Figure 3:**
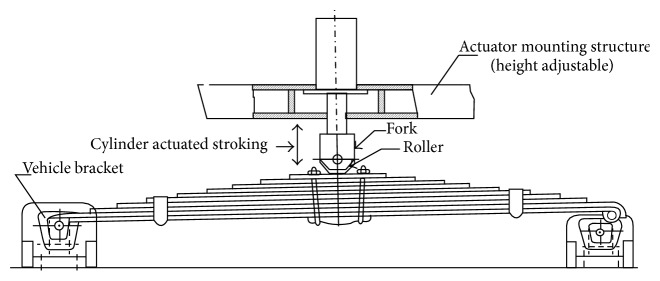
Endurance testing of symmetrical leaf springs [7].

For determination of experimental fatigue life, four specimens (*S*-1, *S*-2, *S*-3, and *S*-4 per batch) for three stress ranges (i.e., 269–896 MPa, 218–969 MPa, and 200–885 MPa) are manufactured with proposed parameters. Similar kind of material processing was done for all the specimens. The stress range was considered for first lot of the specimens to be 627 MPa, 1.3 ± 0.7 g. All the four specimens were tested under same stress range and fatigue life was determined. The stress range for second lot of the specimen was 751 MPa based on SAE spring design manual approach, without considering assembly stresses; that is, all the leaves with common curvature were assembled. The stress range for third lot of the specimen was 685 MPa based on SAE spring design manual approach, by considering assembly stresses. The spring was clamped in the centre to simulate its installation in the vehicle as shown in Figure 3.

As per the requirement specified by the vehicle manufacturer, the leaf springs are to be tested on full scale testing machine as per 1.3 ± 0.7 g. The maximum load will be 2 g and the minimum load will be 0.6 g. Here g represents the design load.

Consider

(16) $$\begin{array}{c} S_{\max}=\frac{P_{\max}*L_{a}*t}{8*\sum I_{\text{total}}}\\ S_{\min}=\frac{P_{\min}*L_{a}*t}{8*\sum I_{\text{total}}}\\ S_{\max}=\frac{25918*1150*8}{8*33230}=896.9\;\text{MPa}\\ S_{\min}=\frac{7775.4*1150*8}{8*33230}=269\;\text{MPa}. \end{array}$$

## 7. Results and Discussion

### 7.1. Experimental Fatigue Life

The experimental fatigue life at different stress range is shown in Table 8. The material processing for all the twelve specimens is the same, that is, normal rolling, quenching at 880°C, tempering at 410°C for 90 mins, shot peening at 18 A intensity, BHN 380–432, and scragging at 0.9% of yield stress. For the first lot of the specimens the maximum stress is 896 MPa and minimum stress is 269 MPa as specified by the vehicle manufacturer. The fatigue life for the four specimens is 84212, 81961, 82226, and 85656 number of cycles. The average stress life is 83513. The second lot of specimen is assembled by considering common curvature, that is, without assembly stresses. The maximum stress is 969 MPa and minimum stress is 218 MPa. The fatigue life for the four specimens is 66796, 69320, 70119, and 69956 number of cycles. The average stress life is 69047. The third lot of specimens is assembled by considering the assembly stresses and leaves with proposed individual leaf camber assembled by pulling against each other to establish common curvature. The fatigue life for the four specimens is 74827, 76658, 79010, and 77229 number of cycles. The average stress life is 76931. It is observed that the stress range for first lot, second lot, and third lot is 627 MPa, 751 MPa, and 685 MPa, respectively. The maximum stress for first lot, second lot, and third lot is 896 MPa, 969 MPa, and 885 MPa, respectively. It is observed that the lower the stress range is the higher the fatigue life will be. But for almost same stress range, the fatigue life decreases by increasing the maximum stress. It is also observed that the experimental fatigue life increases by 11.41% by considering assembly stresses. Other factors like shape, size, temperature, surface, and so forth also affect the fatigue life of the leaf springs which can be considered for estimation of fatigue life [9].

### 7.2. Individual Leaf Camber

**Figure 4:**
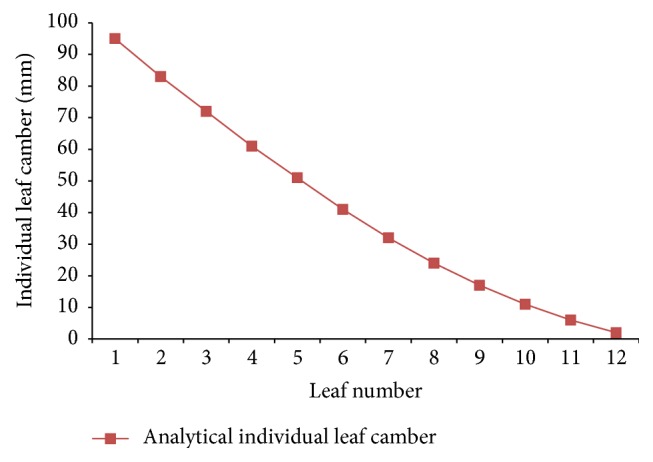
Analytical individual leaf camber.

Figure 4 shows the individual leaf camber for the leaves. It is observed from Figure 4 that the individual leaf camber is 99 mm, which is decreasing from the main leaf. The individual leaf camber is 84 mm for the second leaf with military wrapper. Similarly, the last leaf has individual leaf camber of 2 mm. Hence, the individual leaf camber decreases from the main leaf to the last leaf (almost flat).

### 7.3. Theoretical Fatigue Life Determination

**Figure 5:**
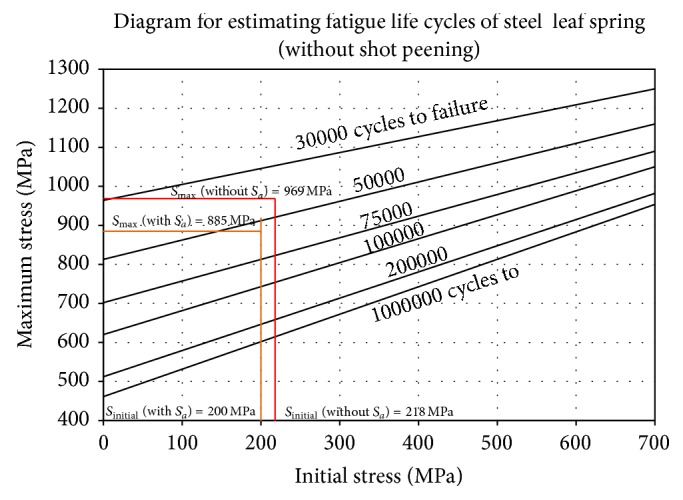
Maximum versus initial stress plot (without shot peening) [8].

From Figure 5 it is observed that the maximum stress induced in the leaf springs without considering the assembly stresses is 969 MPa and the initial stress value is 218 MPa. The intersection of 969 MPa and 218 MPa lies in the zone of 30000 to 50000 cycles. As the point of intersection is nearer to 50000 cycles line therefore it will approximately sustain around 46000 cycles.

It is observed that the maximum stress induced in the leaf springs by considering the assembly stresses is 885 MPa and the initial stress value is 200 MPa. The intersection of 885 MPa and 200 MPa lies in the zone of 50000 to 75000 cycles. As the point of intersection is nearer to 50000 cycles line therefore it will approximately sustain around 58000 cycles. The shot peening and scragging will improve the fatigue life by minimum of 20%. Therefore, fatigue life of leaf springs without and with considering assembly stresses would be approximately (46000∗1.2) 55200 cycles and (58000∗1.2) 69600 cycles, respectively.

### 7.4. Theoretical and Experimental Fatigue Life Comparison

Table 9 shows the fatigue life comparison between the SAE spring design manual and experimental testing, by considering and not considering the assembly stresses. It is observed from Table 9 that as per SAE approach the fatigue life without considering the assembly stresses is 55200 cycles and by considering the assembly stress the fatigue life is 69600 cycles.

As per SAE approach the fatigue life increases by 26.08% by considering assembly stress. The fatigue life is 69047 and 76931 cycles without and by considering the assembly stresses, respectively, in the experimental testing. In experimental testing it is observed that the fatigue life increases by 11.41% due to the reduction in stress range, and maximum stress has reduced from 969 MPa to 885 MPa by considering assembly stress.

## 8. Conclusions

The theoretical and experimental fatigue life of a light commercial vehicle leaf spring is determined by considering assembly stresses and without considering assembly stresses, and following conclusions are made.

1. The maximum stress induced in the leaf spring reduces and uniform stress distribution is achieved by considering the assembly stresses. The fatigue life increases due to negative assembly stresses, which reduces the maximum stress.
2. It is also concluded that for the same stress range, higher maximum stress reduces the fatigue life of the leaf spring and higher initial stress improves the fatigue life of the leaf springs.

## Figures and Tables

**Table 1:** Chemical composition of 65Si7.

Grade	C%	Si%	Mn%	S%	P%	Cr%

65Si7	0.53	0.20	0.72	0.007	0.019	0.73

**Table 2:** Mechanical properties of 65Si7.

Mechanical property	Young's modulus (*E*)	BHN	Poisson's ratio (*μ*)	Tensile strength ultimate (*S*_ut_)	Tensile strength yield (*S*_*y*_)	Elongation at fracture (minimum)	Density (*ρ*)

Value	200124 MPa	380–432	0.266	1272 MPa	1081.2 MPa	7%	0.00000785 kg/mm^3^

**Table 3:** Design parameters of the leaf springs.

Span (*L*) (mm)	1150 ± 3
Load rate (*k*) (N/mm)	159.11 ± 7%
Load (N)	
Rated (P/g)	12959
Maximum (*P*_max⁡_)	28010
No load camber (*C*_*a*_) (mm)	95 ± 4
Seat length (mm)	100
Total number of leaves (*N*)	12
Number of full length leaves (*X*)	2
Maximum thickness of the individual leaf (*t*) × width (*b*), (mm × mm)	8 × 70
Ride clearance (*X*_*c*_) (mm)	94.6
Stiffening factor (SF)	1.1
Required fatigue life (*N*_*f*_) at (1.3 ± 0.7 g)	70000 cycles

**Table 4:** Stress at design load and at maximum load by considering assembly stresses.

Leaf number	Thickness *t* (mm)	Front/rear cantilever stress at design load, *S*_*d*_, (MPa)	Front/rear cantilever stress at maximum load, *S*_*m*_, (MPa)	Assembly stress, *S*_*a*_, (MPa)	Stress at design load with assembly stress, *S*_da_, (MPa)	Stress at maximum load with assembly stress, *S*_ma_, (MPa)	*S*_*a*_ · *t* ^2^
1	8	409.48	885.11	−19	390.48	866.11	−1216
2	8	409.48	885.11	−15	394.48	870.11	−960
3	8	409.48	885.11	−12	397.48	873.11	−768
4	8	409.48	885.11	−10	399.48	875.11	−640
5	8	409.48	885.11	−8	401.48	877.11	−512
6	8	409.48	885.11	−6	403.48	879.11	−384
7	8	409.48	885.11	−5	404.48	880.11	−320
8	8	409.48	885.11	−4	405.48	881.11	−256
9	8	409.48	885.11	−3	406.48	882.11	−192
10	8	409.48	885.11	−2	407.48	883.11	−128
11	8	409.48	885.11	−1	408.48	884.11	−64
12	7	358.30	774.47	111	469.30	885.11	5439
The various assembly stresses chosen provide ∑*S*_*a*_ · *t* ^2^ = −1, which is acceptable.						∑S_a_ · t^2^ =	−1

**Table 5:** Individual leaf camber.

Leaf number	*S*_*a*_ (MPa)	*S*_*a*_/*E*∗*y*	*y* = *t*/2 (mm)	*q*_*n*_ = *q*_*o*_ − *S*_*a*_/(*E*∗*y*) (mm^−1^)	*R*_*l*_ = 1/*q*_*n*_ (mm)	Individual leaf camber (*C*_*l*_) = *q*_*n*_∗*L* ^∧^2/8, (mm)
1	−19	−2.37353*E* − 05	4	0.000598404	1671.110508	99
2	−15	−1.87384*E* − 05	4	0.000593408	1685.182414	84
3	−12	−1.49907*E* − 05	4	0.00058966	1695.892856	80
4	−10	−1.24923*E* − 05	4	0.000587161	1703.109109	69
5	−8	−9.9938*E* − 06	4	0.000584663	1710.387036	56
6	−6	−7.49535*E* − 06	4	0.000582165	1717.727431	44
7	−5	−6.24613*E* − 06	4	0.000580915	1721.421307	34
8	−4	−4.9969*E* − 06	4	0.000579666	1725.131104	23
9	−3	−3.74768*E* − 06	4	0.000578417	1728.856925	17
10	−2	−2.49845*E* − 06	4	0.000577168	1732.598874	11
11	−1	−1.24923*E* − 06	4	0.000575918	1736.357057	6
12	111	0.000158473	3.5	0.000416196	2402.714037	2

**Table 6:** Analytical results for load, deflection, and bending stress.

Serial number	Load type	Load (N)	Deflection (mm)	Bending stress by total length (MPa)	Bending stress by active length (MPa)
1	Unladen load	7661	50.3	265.2	242
2	Design/rated load	12959	84.6	448.48	409
3	Flat load	15754	102.8	545.2	498
4	Rubber touching load	21645.7	141.37	749.08	684
5	Metal-to-metal contact	28010	182.9	969.2	885

**Table 7:** Experimental results for load, deflection, and bending stresses.

Serial number	Load type	Load (N)	Deflection (mm)	Bending stress (MPa)
1	Unladen load	7661	46.9	262
2	Design/rated load	12959	81.44	446
3	Flat load	15754	99	540
4	Rubber touching load	21645.7	136	743
5	Metal-to-metal contact	28010	176	941

**Table 8:** Experimental fatigue life of the specimens under different stress ranges.

Material processing	Standard specified	Alternating stress level (MPa)	Stress range	Fatigue life of *S*-1 (*N*_*f*_)	Fatigue life of *S*-2 (*N*_*f*_)	Fatigue life of *S*-3 (*N*_*f*_)	Fatigue life of *S*-4 (*N*_*f*_)	Average SD
Normal rolling, quenching at 880°C, tempering at 410°C for 90 mins, shot peening at 18 A intensity, BHN 380–432, and scragging at 0.9% of yield stress	0.6 g–2 g (As specified)	269–896	627	84212	81961	82226	85656	Avg. 83513 SD 1518

Normal rolling, quenching at 880°C, tempering at 410°C for 90 mins, shot peening at 18 A intensity, BHN 380–432, and scragging at 0.9% of yield stress	(Without *S*_*a*_)	218–969	751	66796	69320	70119	69956	Avg. 69047 SD 1333

Normal rolling, quenching at 880°C, tempering at 410°C for 90 mins, shot peening at 18 A intensity, BHN 380–432, and scragging at 0.9% of yield stress	(With *S*_*a*_)	200–885	685	74827	76658	79010	77229	Avg. 76931 SD 1492

**Table 9:** Fatigue life comparison between SAE approach and experimental result.

Serial number	SAE spring design manual approach		% age variation	Experimental testing		% age variation
	Fatigue life without assembly stress, *S*_*a*_	Fatigue life with assembly stress, *S*_*a*_		Fatigue life without assembly stress, *S*_*a*_	Fatigue life with assembly stress, *S*_*a*_	
1	55200	69600	26.08%	69047	76931	11.41%

## Nomenclature

*C*_*a*_: No load assembly camber
*C*_*l*_: Individual leaf camber
*E*: Young's modulus of elasticity
*N*_*f*_: Number of cycles to failure
*q*_*n*_: Individual leaf curvature
*q*_*o*_: No load assembly curvature
*R*_*a*_: No load assembly radius
*R*_*l*_: Free radius for individual leaf
*S*_*a*_: Assembly stress
*S*_*d*_: Stress at design load without assembly stress
*S*_da_: Stress at design load with assembly stress
SF: Stiffening factor
*S*_fa_: Stress on the front cantilever
*S*_*l*_: Load stress
*S*_*m*_: Stress at maximum load without assembly stress
*S*_ma_: Stress at maximum load with assembly stress
*S*_rb_: Stress on the rear cantilever
*S*_*t*_: Total stress
*S*_ut_: Tensile strength ultimate
*S*_*y*_: Yield strength
*X*_*c*_: Ride clearance
*ρ*: Density
*μ*: Poisson's ratio.
